# Long-Term Health-Related Quality of Life (HRQoL) After Redo-Fundoplication

**DOI:** 10.1007/s00268-021-05954-3

**Published:** 2021-01-27

**Authors:** Antti J. Kivelä, Juha Kauppi, Jari Räsänen, Anna But, Harri Sintonen, Jaana Vironen, Olli Kruuna, Tom Scheinin

**Affiliations:** 1 Department of GI Surgery, Abdominal Centre, Helsinki University Hospital and Helsinki University, Jorvi Hospital, Turuntie 150, P.O. Box 800, FI 00029 Espoo, Helsinki, HUS Finland; 2 Department of General Thoracic and Esophageal Surgery, Lung and Heart Center, Helsinki University Hospital and Helsinki University, Helsinki, Finland; 3 University of Helsinki, Helsinki, Finland; 4 Department of Public Health, Faculty of Medicine, University of Helsinki, Helsinki, Finland

## Abstract

**Background:**

We aim to shed light on long-term subjective outcomes after re-operations for failed fundoplication.

**Methods:**

1809 patients were operated on for hiatal hernia and/or gastroesophageal reflux disease (GERD) at the Helsinki University Hospital between 2000 and 2017. 111 (6%) of these had undergone a re-operation for a failed antireflux operation. Overall, HRQoL was assessed in 89 patients at the latest follow-up using the generic 15D© instrument. The results were compared to a sample of the general population, weighted to reflect the age and gender distribution of patients. Disease-specific HRQoL was assessed using the GERD-HRQoL questionnaire. We studied variation in the overall HRQoL with respect to disease-specific HRQoL and known patients' parameters using univariate and multivariable linear regression models.

**Results:**

The median postoperative follow-up period was 9.3 years. All patients were operated on laparoscopically (6% conversion rate), and 87% were satisfied with the re-operation. Postoperative complications were minimal (5%). Twelve patients (11%) underwent a second re-operation. The median GERD-HRQoL score was nine. In multivariable analysis, four variables were independently associated with the 15D score, suggesting a decrease in the 15D score with increasing GERD-HRQoL score, increasing Charlson Comorbidity Index (CCI) and the presence of chronic pain syndrome (CPS) and depression.

**Conclusion:**

Re-do LF is a safe procedure in experienced hands and may offer acceptable long-term alleviation in patients with recurring symptoms after antireflux surgery. Decreased HRQoL in the long run is related to recurring GERD and co-morbidities.

## Introduction

Re-operation rate has been reported between 3 and 6% after failed laparoscopic fundoplication (LF) and most commonly due to recurrent reflux and dysphagia [1, 2]. Short-term objective outcome and patient satisfaction have been reported to be good, although results are not comparable to that of primary LF [3]. Less is known about health-related quality of life (HRQoL) related to re-do fundoplication. Short-term improved disease-specific [4] and global quality of life after re-fundoplication has been reported [5, 6]. Decreased HRQoL as compared to that of primary LF [7, 8] or general population [9] after re-fundoplication is reported by at least three studies. In this study, we aimed to shed light on patient satisfaction, disease-specific and generic HRQoL, and factors associated with the generic HRQoL of patients who had undergone re-do LF.

## Materials and methods

### Patients

We identified 1809 patients who had had anti-reflux surgery over the period 2000–2017 at Helsinki University Hospital. Clinical details were reviewed from medical records. Informed consent was obtained from selected patients, and two quality of life instruments, the 15D and GERD-HRQoL, were sent. Also, patients were asked if they were satisfied with the outcome and if they would undergo a second operation again. This study was approved by the Ethics Committee of Helsinki University Hospital. According to the hospital database, 111 (6%) had had redo anti-reflux surgery. Of these 111 patients, 89 (87%) participated in the survey for an evaluation of their QoL. Of the 22 non-responding patients, six had died of causes non-related to antireflux surgery, and seven patients’ records were unavailable. Two patients were unable to answer due to acquired cognitive deficits during the follow-up. Preoperative workup consisted of esophagogastroscopy, barium swallow and computed tomography, to identify possible anatomic failures causing the symptoms. Patients were presented to us through referrals or direct contact due to recurring or new symptoms. Patients were considered for surgery if correlation of symptoms and repairable anatomic failure was present, and medication or dietary counseling was not beneficial. In selected patients, impedance measurement, manometry and scintigraphic emptying studies were also performed. Patient data were collected, including demographics, BMI, surgical techniques, medications, perioperative morbidity and complications, hospitalization, time to recurrence and follow-up. Depression or chronic pain syndrome was considered present if patient records revealed use of antidepressants or chronic pain medication, or diagnosis of depression or a chronic pain-causing condition.

### Operative technique

The first laparoscopic port is inserted away from any previous incisions. After adhesiolysis, the other 3 ports and liver retractor are placed in typical positions for LF. Either a 5 mm or 10 mm camera is used, based on the surgeon’s preference. The constant flow of ${CO}_{2}$ at pressure of 12 mm Hg is used for insufflation. Ultrasonic coagulating shears are used. The complete take down of previous repair is necessary to fully assess the cause of failure. Both crura are freed from scars, and care is taken to preserve the peritoneum covering the crura and the integrity of the crura. Mobilization of the esophagus into the mediastinum is performed to achieve at least 3 cm of free esophagus in the abdomen.

A floppy 2- to 3-cm 360-degree Nissen wrap is performed over the esophagus with a 45-54fr Maloney dilator or 32fr orogastric tube in place for calibration, according to the surgeon’s preference. Partial wraps were used in selected cases. The cruras are approximated both posterior and anterior to the wrap, avoiding any threshold formation. If there is significant tension in crural re-approximation, permanent (Cousin® and Crurasoft®) or biosynthetic resorbable (Gore® Bio-A® and VERITAS®) mesh is used.

### Questionnaires

#### Generic HRQoL analysis

HRQoL was measured by the 15D. This is a generic, 15-dimensional, standardized, self-administered instrument that can be used both as a profile and as a single index score measure. The health state descriptive system (questionnaire) is composed of the following dimensions: mobility, vision, hearing, breathing, sleeping, eating, speech (communication), excretion, usual activities, mental function, discomfort and symptoms, depression, distress, vitality and sexual activity. For each dimension, the respondent chooses one of the five ordinal levels best describing his/her state of health at the time (best level = 1; worst = 5).

The valuation system is based on an application of the multi-attribute utility theory. The single index score (15D score), representing the overall HRQoL on a 0–1 scale (1 = full health, 0 = being dead), and the dimension level values, reflecting the goodness of the levels relative to no problems on the dimension (= 1) and to being dead (= 0), are calculated from the health state descriptive system using a set of population-based preference or utility weights. Mean dimension level values are used to draw 15D profiles for groups [10]. The minimum clinically important change or difference in the 15D score has been estimated to be ± 0.015 on the basis that patients can feel such a difference on average [11].

The data for the general population came from the National Health 2011 Health Examination Survey, representing the Finnish population aged 18 and over [12]. For this analysis, individuals who fell in the age range of patients in the catchment area of the Helsinki University Hospital (n = 1178) were selected. This population sample was weighted to reflect the age and gender distribution of patients.

#### GERD-HRQoL

The GERD-HRQoL questionnaire evaluates heartburn, dysphagia and regurgitation during daily life on a scale from zero (no symptoms) to five (incapacitating symptoms) in 15 questions. Maximum score ranges from zero to 75 points [1].

### Statistical analysis

The main outcome parameter was the 15D score. Descriptive statistics are presented as means with standard deviations (SD) or medians and interquartile range (IQR) for continuous data, and frequencies and proportions (%) for categorical data. We used mean for 15D, to make comparisons with sample population and other QoL studies possible. Otherwise, medians were reported. Independent samples t-test was used to assess the statistical significance of the differences in the mean 15D score and each of its 15 dimensions between the patients and the general population. Linear regression analysis was performed to identify patient characteristics associated with the 15D score. Based on *p* < 0.2 in the univariate linear regression analysis, we selected candidate variables to be considered in the multivariable analysis. We constructed the final model by entering these variables one by one and by retaining those that were associated with the 15D score (*p* < 0.05). We inspected goodness-of-fit of the final model as appropriate, including visual examination of the residuals and assessment of multicollinearity and unusual and influential data. We detected four observations that were both outliers and influential. After removing these observations, we refitted the final model and found the model to fit the data satisfactorily. The analyses were performed on SPSS version 22. (IBM, Armonk, NY).

## Results

### Demographics, operative findings and cumulative failure rates

Patient characteristics are shown in Table 1. Of the non-respondents, those with patient records available (*N* = 9) were similar to the responders with respect to their age and CCI distributions (mean age 61 years, and mean CCI score 3). The complications during and after the operation are shown in Table 2. Total intraoperative complication rate was 4%. There was no postoperative mortality, and total postoperative complication rate was 5%, all graded less than Clavien–Dindo grade 3 [13]. During the follow-up, re-recurrent hiatal hernia was detected in 18% (*n* = 16) of the patients; a re-reoperation was performed in 12% (*n* = 11). Five patients with re-recurrent hiatal hernia were not re-operated on for various reasons. Meshes were used in 19 of 89 first time redos (21%). In spite of the mesh, six of these 19 (32%) needed a second redo operation. All but two of the first re-do operations were laparoscopies, the exceptions being left-sided thoracotomies for strangulated paraesophageal hernias. In six of the 11 patients (27%) who underwent a second re-do operation, left thoracotomy was performed for strangulated hernias, and three of them required an esophageal resection with later reconstruction.

**Table 1:** Patient characteristics of the eighty-nine redo-fundoplication patients at the time of the first re-operation

N = 89	MEAN(SD)/MEDIAN(IQR) / NUMBER(%)
AGE at the time of questionnaires	Mean 61 years (SD 11.1) Median 63 (IQR 48, 61)
Time since first re-do operation	Mean 8.3 years (SD 3.9) Median 9.3 (IQR 5.3, 11.9)
GENDER, MALE	38 (43%)
BMI (redo)	Mean 26.6 (SD 4.3) Median 27 (IRQ 24, 30)
Using PPI at the time of questionnaires	Yes 56 (63%) No 33 (37%)
CCI score	28 (32%) 2–4 39 (45%) > 4 20 (23%)
Depression	Yes 26 (29%) No 63 (71%)
CPS	Yes 23 (27%) No 66 (73%)
Pulmonary disease	Yes 14 (16%) No 75 (83%)
PRIMARY OPERATION	Nissen = 83 Toupet = 6
SYMPTOMS FOR RE-OPER	Heartburn/regurgitation = 57 Dysphagia = 14 Heartburn/regurgitation and dysphagia = 11 Other = 6
ANATOMIC FAILURE	Hiatal hernia (Type I) = 44 Hiatal hernia (Type III, acute) = 2 Disrupted fundoplication = 13 Misplaced fundoplication = 23 Twisted fundoplication = 3 Other = 4
RE-OPERATION	Nissen = 83 Toupet = 6
BLEEDING	MEDIAN = 0 ml (IQR 0, 42.5)
MESH	19 (21%)

BMI—Body mass index; PPI—proton pump inhibitor; CCI—Charlson comorbidity index; CPS—chronic pain syndrome

**Table 2:** Intra- and postoperative complications

INTRAOPERATIVE COMPLICATION	N = 89
Perforation	2 (2%)
Pneumothorax	1 (1%)
Bleeding	1 (1%)
POSTOPERATIVE COMPLICATION (30d)	
Pneumonia	1 (1%)
Ileus	1 (1%)
Pulmonary embolism	2 (2%)
Fever	1 (1%)

### Symptom Resolution

At the latest follow-up, 77 (87%) reported satisfactory outcomes. Recurrent reflux as the primary complaint (*n* = 72) resolved in 82%. Dysphagia as the primary complaint (*n* = 24) resolved in 67%. Continuous PPI use was reported by 39% and occasional use in 24%. Given the benefit of hindsight, 79% of all patients would have the operation performed again.

### Self-Reported HRQoL

**Fig. 1:**
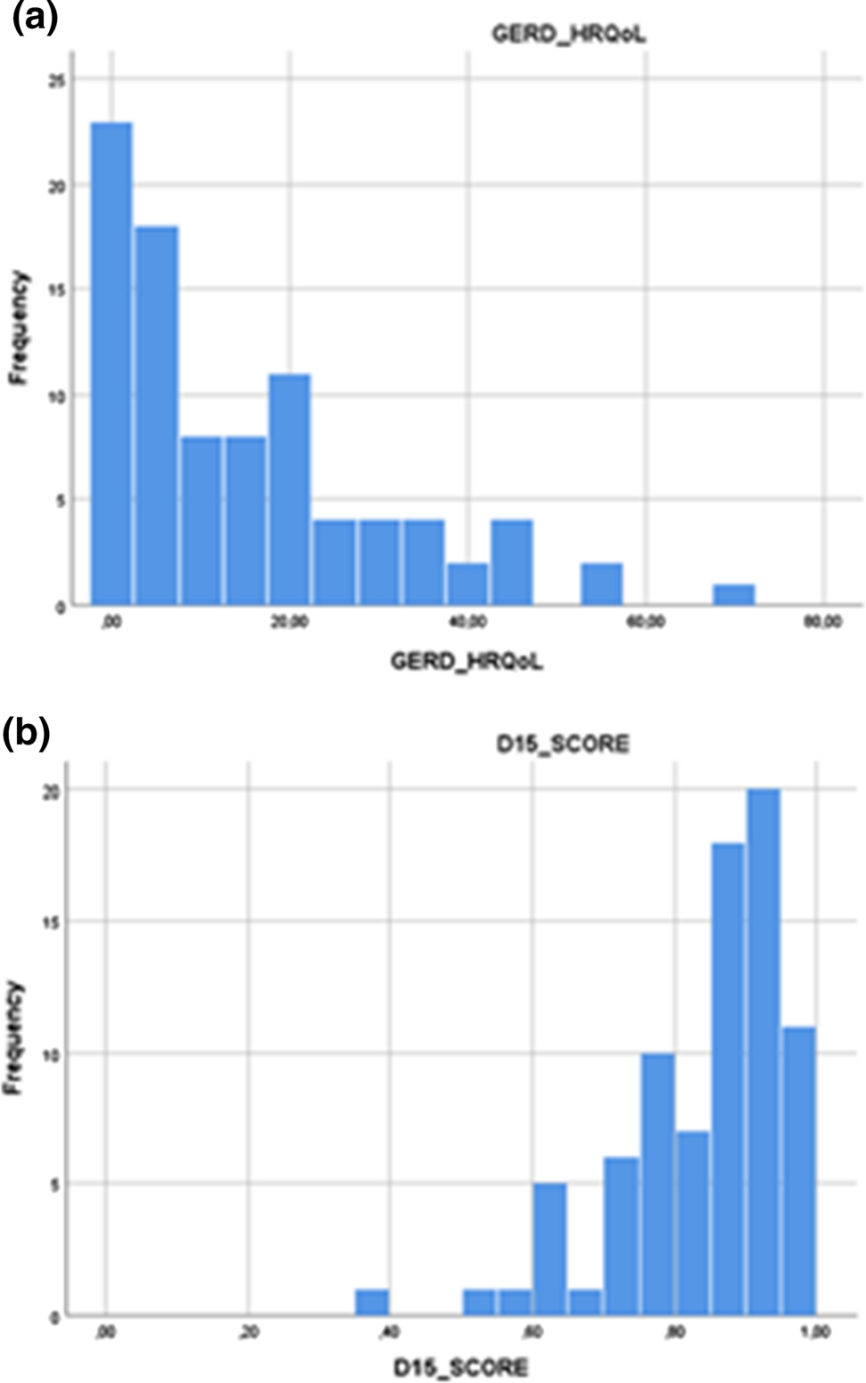
**a)** Distribution curve of scores of GERD-HRQoL questionnaire. The questionnaire gains values between 0 and 75. Zero points means no symptoms and 75 sever and disabling symptoms. Median score was 9 (IQR 2, 20). **b)** Distribution curve of scores of the 15D-HRQoL questionnaire. The score is a composite of all 15 dimensions and gets values between 0 and 1. Higher values reflect better HRQoL. Mean 15D score was 0.85 (SD 0.12)

**Fig. 2:**
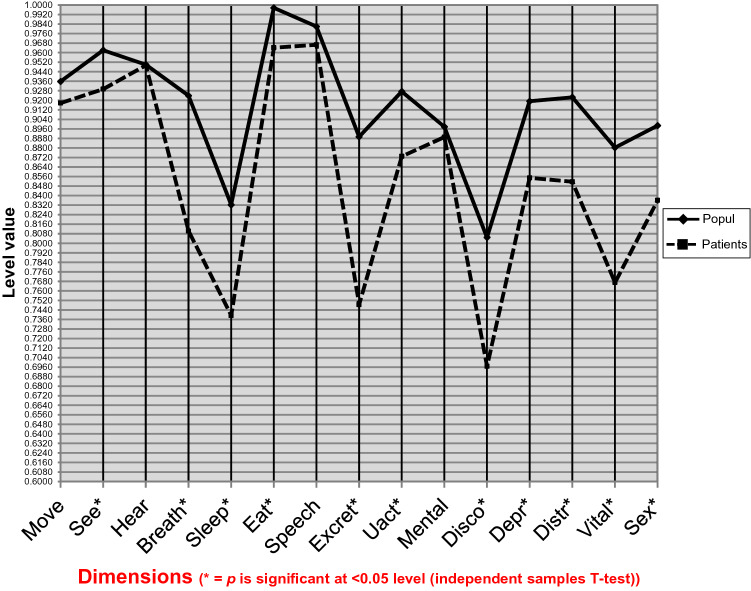
The mean 15D profile of the study population compared to that of the age- and gender-standardized general population. The data for the general population came from the National Health 2011 Health Examination Survey representing the Finnish population aged 18 and over [12]. For this analysis, those individuals were selected, who were in the age range of patients in the catchment area of the Helsinki University Hospital (*n* = 1178). This sample was weighted to reflect the age and gender distribution of patients. Move—mobility; Excret—excretion; UACT—usual activities; Mental—mental function; Disc—discomfort and symptoms; Depr—depression; Distr—distress; Vital—vitality; Sex—sexual activity. *p is significant at < 0.05 level (independent samples T-test)

The mean 15D score of was 0.854 (SD 0.124) and median GERD-HRQoL 9 (IQR 2, 20) (Fig. 1a, b). The mean 15D score was lower than in a sample of the general population of similar age and sex distribution. (0.854 vs 0.915, *p* < 0.001). The total 15D score had a statistically significant linear association with the GERD-HRQoL score (Pearson correlation *r* = −0.510, *p* < 0.001), and separate dimensions of the 15D questionnaire were significantly correlated with GERD-HRQoL, in all except dimensions for hearing and mental function (Table 3). There were statistically significant differences between the patients and the general population with regard to the 15D dimensions (Fig. 2), in all but four dimensions (moving, hearing, speech, mental function). We observed low GERD-HRQoL values (Fig. 1a) in the majority of the patients, the median being nine (IQR 2–20). In the univariate analysis, a statistically significant association was seen between the 15D score and several continuous and dichotomous variables (Table 4), including a negative association with GERD-HRQoL score (*p* < 0.001), the presence of depression (*p* = 0.001) and CPS (< 0.001). We observed a decrease in the 15D score with increasing GERD-HRQoL score, increasing CCI score and the presence of chronic pain syndrome (CPS) and depression (Table 5). Together, these variables explained 52.7% of the variation in the 15D score.

**Table 3:** Pearson correlation coefficients (*r*) and corresponding p-values between GERD-HRQoL and dimensions of 15D (*N* = 89)

Correlations		
		GERD-HRQoL
SEE	R	−236
	*P*	0.026
HEAR	R	−035
	*P*	0.748
BREATH	R	−428
	*P*	< 0.001
MOVE	R	−311
	*P*	0.003
SLEEP	R	−331
	*P*	0.002
EAT	R	−388
	*P*	< 0.001
SPEECH	R	−413
	*P*	< 0.001
EXCRET	R	−415
	*P*	< 0.001
UACT	R	−320
	*P*	0.002
MENTAL	R	−083
	*P*	0.440
DISCO	R	−526
	*P*	< 0.001
DEPR	R	−326
	*P*	0.002
DISTR	R	−244
	*P*	0.021
VITAL	R	−383
	*P*	< 0.001
SEX	R	−385
	*P*	< 0.001

GERD-HRQoL—Gastroesophageal reflux disease health-related quality of life; r—Pearson correlation; Move—mobility; Excret—excretion; UACT—usual activities; Mental—mental function; Disc—discomfort and symptoms; Depr—depression; Distr—distress; Vital—vitality; Sex—sexual activity

**Table 4:** Regression coefficients and their 95% confidence intervals and p-values (t-test) from the univariate linear regression models for the 15D score as the dependent variable (N=89)

	Regression coefficient	95% CI	*p*-value
Age at latest FUP	−0.001	−0.003, 0.001	0.411
CCI	−0.009	−0.022, 0.004	0.179
GERD-HRQoL	−0.004	−0.006, -0.003	< 0.001
Depression, yes vs. No	−0.097	−0.151, −0.044	0.001
CPS, yes vs. No	−0.131	−0.184, −0.078	< 0.001
Pulmonary disease	−0.014	−0.086, 0.058	0.708
Time since re-operation	−0.001	−0.006, 0.008	0.735
Time to second operation	−0.001	−0.004, 0.003	0.764
BMI	−0.003	−0.009, 0.003	0.293
Gender, men vs. women	0.034	−0.018, 0.087	0.198
PPI at late FUP, yes vs no	−0.074	−0.126, −0.022	0.006
Recurrent HH, yes vs. no	−0.033	−0.101, 0.035	0.341
Second re-do, yes vs. no	−0.038	−0.117, 0.042	0.347

GERD-HRQoL—Gastroesophageal reflux disease Health-Related Quality of Life Questionnaire; CPS—chronic pain syndrome; CCI—Charlson comorbidity index; BMI—body mass index; PPI—proton pump inhibitor; FUP—follow-up; HH—recurrent hiatal hernia

**Table 5:** Regression coefficients as fitted to the data of 85 patients and their 95% confidence intervals (CI) and p-values (t-test) from the final multivariable linear regression model for the 15D score as the dependent variable. Adjusted R-square 0.527

	Unstandardized coefficients (B)	Standardized coefficients (Beta)	95% CI for B	*p*-value
CCI score	−0.016	−0.225	−0.025, −0.007	< 0.001
GERD-HRQoL	−0.004	−0.500	−0.005, −0.003	< 0.001
Depression	−0.050	−0.187	−0.091, −0.008	0.019
CPS	−0.090	−0.324	−0.133, −0.046	< 0.001

GERD-HROoL—Gastroesophageal reflux disease Health-Related Quality of Life Questionnaire; CPS—chronic pain syndrome; CCI—Charlson comorbidity index

## Discussion

We found that although the majority of patients after re-do LF remained satisfied in the long-term follow-up, overall HRQoL of the study group patients was lower than that of an age- and gender-standardized sample of the general population. Lower overall HRQoL was associated with higher GERD-HRQoL and CCI scores and the presence of depression and CPS.

The 15D instrument was chosen to measure HRQoL, as it has been validated in the Finnish general population, making it suitable for our comparisons [14]. To quantify severity of GERD, GERD-HRQoL was used, which is a commonly used disease-specific instrument [15] and is known to have an association with both disease-specific and generic HRQoL [3, 16]. Statistically significantly lower 15D-scores were seen among patients with worse GERD-HRQoL scores in this study as well. As GERD-HRQoL score was also correlated with most dimensions of 15D, the severity of GERD symptoms is likely the most important reason explaining the difference of HRQoL between the study group and general population. As the mean age of our cohort is 61 years, it cannot be ruled out that depression, CPS and diseases included in CCI [17] also affect HRQoL in the general population. However, the mean 15D depression (12th dimension) scores of the study population were significantly lower than in the general population, suggesting that depression plays a role in explaining the difference. Also, as our study group represents a highly selected group of complicated GERD patients, similar patients are unlikely to be found in the general population [18]. To our knowledge, the effect of co-morbidity on global HRQoL after a re-fundoplication has not been studied, whereas depression [19–21], somatoform syndromes [22] and chronic pain syndrome (CPS) [23, 24] have been associated with poor HRQoL after primary antireflux surgery. These studies support our findings, CPS and depression being associated with lower global HRQoL in GERD patients who have undergone a redo-fundoplication.

The median GERD-HRQoL of nine (IQR 2, 20) in our patients is comparable to similar studies [25], suggesting satisfactory reflux control in our patients. More than half of our patients were on PPIs in the long-term follow-up, which is slightly more than in previous reports [26]. The use of PPIs probably over-estimates the true incidence of acid reflux, as it is known that use of PPIs and measured esophageal acid exposure do not correlate well, and patients are likely to use PPIs for several causes of dyspepsia [27]. Dallemagne et al. [21] reported results of 144 patients having undergone re-do LF with a mean follow-up of 75.8 months. The global GIQLI score was significantly lower in patients after a re-do operation than in the general population, a result similar to ours. To our knowledge, only two studies report symptomatic outcome with follow-up as long as ours [10, 21]. In series by Oelschlager et al., heartburn was relieved in 61% and dysphagia in 74% of patients. Overall, symptomatic success rate was 68%. In a study by Dallemagne et al. 73% of patients operated for reflux and 68% of patients operated for dysphagia reached resolution of symptoms. Our results of primary symptom resolution compare well with these studies.

Our rates of intra- (3%) and postoperative (6%) complications are less than reported in the literature [2, 28]. In addition, there were no complications classified over Clavien–Dindo grade 2B [13]. There was a recurrent hiatal hernia in 16 (18%) of our patients, with increasing cumulative risk of failure during follow-up. Eleven patients with recurrent hiatal hernia underwent second re-operation. Recurrent hiatal herniation was not associated with lower scores in the 15D-questionnaires. Dallemagne et al. also report incidence of failure after re-do increasing with time, and 41% risk of failure in repair of hiatal herniation [21]. Our results are comparable. Three patients of our series (3%) had to undergo esophageal resection with reconstruction as a third operation. Rate of resection is the same in a review by Furnée et al. [2] that found a 2.7% rate of esophageal resections.

The strength of this study is the long-term follow-up of patients after re-do LF. The majority of the patients could be reached for follow-up. Furthermore, experienced surgeons of the same team selected the patients for re-operation and carried out the operations. A major limitation is the retrospective nature and lack of control group of GERD patients and questionnaires for HRQoL before re-do LF.

Our results suggest that patients with multiple co-morbidities, CPS and depression should be counseled and optimized for underlying conditions before re-do LF. As depression, cps and co-morbidities did seem to decrease HRQoL of our patients, so did increasing severity of heartburn and regurgitation. Given the complex nature of symptoms in this group of patients and very long follow-up, the results of the GERD-HRQoL and 15D HRQoL questionnaires can be considered acceptable. Patient selection plays a crucial role: when symptoms and anatomic failure match, surgery may offer long-term relief and patients with psychiatric conditions should not be denied that possibility. If that is not the case, efforts should be made to continue with conservative options.

In conclusion, re-do LF is a safe procedure in experienced hands and may offer acceptable long-term alleviation in patients with recurring symptoms after antireflux surgery. Worse HRQoL in the long run is related to recurring GERD and co-morbidities.
